# The Use of *Pythium oligandrum* in the Biological Control of Roundworm Infection in Dogs and Cats

**DOI:** 10.3390/pathogens11030367

**Published:** 2022-03-17

**Authors:** Iasmina Luca, Marius Stelian Ilie, Tiana Florea, Adrian Olariu-Jurca, Adrian Stancu, Gheorghe Dărăbuş

**Affiliations:** 1 Department of Parasitology and Dermatology, Banat’s University of Agricultural Sciences and Veterinary Medicine “King Michael I of Romania” from Timisoara, Calea Aradului 119, 300645 Timisoara, Romania; marius.ilie@fmvt.ro (M.S.I.); tijana.florea@usab-tm.ro (T.F.); gheorghe.darabus@fmvt.ro (G.D.); 2 Department of Pathological Anatomy and Forensic Medicine, Banat’s University of Agricultural Sciences and Veterinary Medicine “King Michael I of Romania” from Timisoara, Calea Aradului 119, 300645 Timisoara, Romania; adrian.olariu-jurca@usab-tm.ro (A.O.-J.); astancu2002@yahoo.com (A.S.)

**Keywords:** oomycete, ascarids, pets, ovicidal action, decontamination

## Abstract

*Pythium oligandrum* is an oomycete used in veterinary medicine to treat dermatophytosis in animals. The ovicidal potential against various types of parasite eggs has not been investigated. Ascarids are very common in young animals and the resistance of eggs in the external environment is very high. A commercial product containing *P. oligandrum* was used in the present study. Its ovicidal action against *Toxocara* spp. eggs was investigated. Thus, two categories of media (soil and sandstone) were used, on which the ascarid eggs were dispersed. The commercial product was prepared as a solution and was applied according to the manufacturer. The treatment scheme indicated in case of animals with dermatophytosis was used. Thus, the external natural conditions were recreated in the laboratory, in order to establish whether there is any applicability for this oomycete in the biological control of some parasitic diseases. The results indicated an ovicidal action of *Pythium oligandrum*, supporting the prospects of its use in the decontamination methods of various surfaces or environments where ascarid eggs from carnivores are found.

## 1. Introduction

Human toxocarosis is caused by larvated eggs of the genus *Toxocara*. A major source of human infestation is represented by soil. The survival of parasitic forms depends on the type of soil. Their resistance is increased at pore level in the deeper layers [1,2]. Sandy and friable soils are more aerated, ensuring optimal growth conditions for various eggs or parasite larvae [3]. Ascarid eggs are resistant to various physical and chemical factors due to the internal structure of the wall. The use of nematophagous fungi can be an effective way of controlling these parasites, with a minimal negative impact on the ecosystem. Numerous saprotrophic fungi such as *Chrysosporium merdarium*, *Fusarium oxysporum*, *Fusarium moniliforme*, and *Fusarium sulphureum* [4] have a high ovicidal action. In the case of *Toxocara canis* eggs, *Purpureocillium lilacinum* has inhibitory potential on their viability [5]. *Duddingtonia flagrans* and *Pochonia chlamydosporia* were also used as chlamydospores to control roundworm infection. They show larvicidal action against L2 of *Toxocara canis* due to the extracellular enzymes released and due to the predatory action performed (catching and strangling the larva inside the egg) [6].

Another fungus commonly used in veterinary medicine is *Pythium oligandrum*. It is part of the ingredients of the commercially available product Ecosin^®^ (Maravet, Ostrava, Czech Republic), which is used in the treatment of ringworm infection. The product contains oospores of *Pythium oligandrum*.

The nematophagous action of *Pythium oligandrum* has not been tested so far; therefore, the present study aims to investigate the potential ovicidal action of *Pythium oligandrum* on *Toxocara canis* and *Toxocara cati* eggs, which have been distributed on different soil types. These categories of soil are also commonly found in dog pens. Thus, the natural conditions from the external environment were reproduced in the laboratory. This study evaluates the possibility of using solutions containing *Pythium oligandrum* as dog pen disinfectants, in the control of roundworms in carnivores.

## 2. Results

The ovicidal effect on *Toxocara canis* eggs was confirmed in only three of the five investigated soils, respectively: sandy-clayey clay, medium sandy clay, and medium clay (Table 1). Insignificant differences were present in the coarse sandy clay and loamy fine sand samples (Table 1).

The differences between the percentages of the degenerated eggs in treated and control samples were very significant for sandstone media (Table 1).

The ovicidal effect on *Toxocara cati* eggs was confirmed in all the five types of soil and on sandstone media (Table 2).

**Figure 1:**
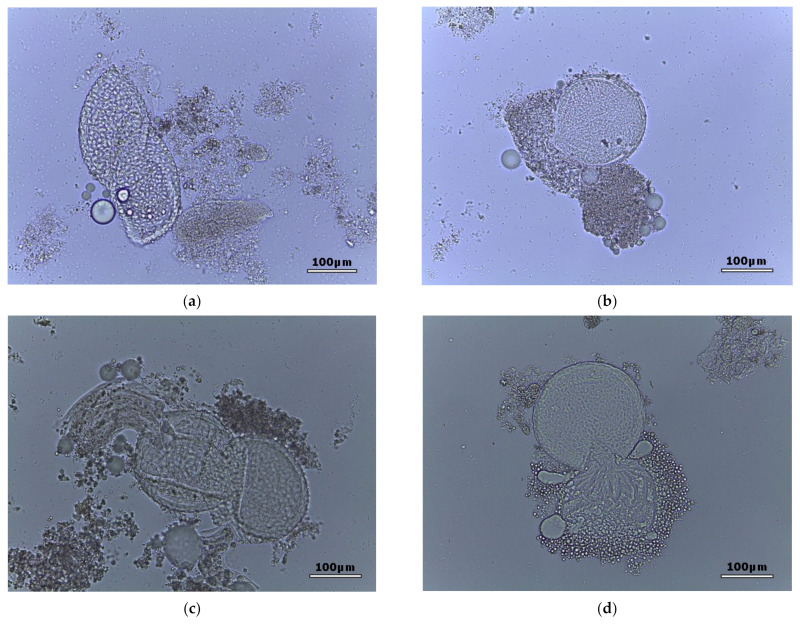
(**a**,**b**) *Toxocara cati* eggs with degenerate internal content and cell wall; (**c**,**d**) *Toxocara canis* eggs with degenerate wall and displaced internal content.

Among the morphological changes induced by the oomycete, there are degenerations of the egg wall along with internal content lysis or extravasation (Figure 1).

**Figure 2:**
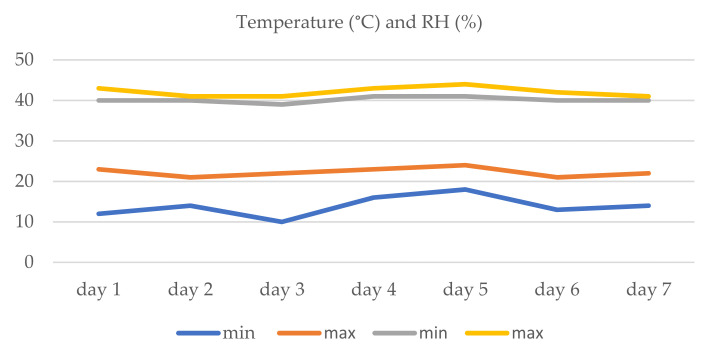
The variations of the temperature (blue and orange) and the relative humidity (RH) (gray and yellow) recorded during the study.

Temperature and humidity are two important factors that influence the development of *Toxocara* spp. eggs. These were monitored throughout the study period (Figure 2). The lowest temperature was recorded on the first day (12 °C), and the highest was reported on the fifth day (24 °C) (Figure 2). The relative humidity, during the seven days, was between 39–44% (Figure 2).

## 3. Discussion

Soil texture and its components are factors that influence the development and viability of parasitic elements. In the case of roundworms, studies indicate that heavy colloidal soils provide the best conditions for survival and resistance. Sandy soils do not retain enough water and thus have a negative effect on the viability of roundworm eggs [3,7,8]. However, some researchers claim that sandy and friable soils are more aerated and, thus, create optimal developmental conditions for various parasite eggs or larvae [9,10].

The obtained data certify the nematophagous, ovicidal potential of *Pythium oligandrum*. Ecosin^®^ contains oospores of this oomycete. Once applied to the media with ascarid eggs, the hyphae begin to germinate. The hyphae adhere to the egg wall and gradually release enzymes. The wall of roundworm eggs is degraded by the kinases, cellulases, endo-β-1,3-glucanases, and various exoglycosidases [11,12]. Moreover, the cell wall is hydrolyzed due to proteases. Thus, the oomycete penetrates inside the eggs and can use the internal content as a nutritious substrate [11,12]. This facilitates the germination of several hyphae from the same host. A zoosporangium is formed in the terminal portion of each hyphae, which releases a large number of zoospores. These zoospores attach to other roundworm eggs at the level of the wall and, thus, begin the germination of new hyphae. The role of *Panicum miliaceum* as an ingredient in Ecosin^®^ is its preservative due to its antioxidant properties [13]. Park et al. (2011) investigated the role of *P. miliaceum* extract on adipocytes and found that it led to a transformation of monounsaturated fatty acids into saturated fatty acids [14]. Since the wall of *Toxocara* spp. eggs contains a layer of phospholipids, composed of saturated and unsaturated fatty acids, following the application of Ecosin^®^, *P. miliaceum* could have influenced the ratio of these acids, modifying the permeability of the wall, thus favoring the penetration and the internal multiplication of the oomycete. Therefore, we can consider that the association with *P. oligandrum* is beneficial for shortening the period of multiplication of the oomycete on a nutritious substrate, respectively the egg wall of *Toxocara* spp.

No similar studies have been identified in the literature, but the action of other species of fungi against eggs and larvae of roundworms has been investigated. Spores of species such as *Mucor circinelloides*, *Paecilomyces lilacinus* and *Verticillium* sp. have a high ovicidal action against *Baylisascaris procyonis* eggs in raccoons [9]. Other authors have observed a reduction in the viability of *Toxascaris leonina* and *Trichocephalus* spp. eggs, after spraying them with solutions containing spores of *Mucor circinelloides*, *Trichoderma atrobrunneum*, or *Verticillium* sp. [10]. Ovistatic and ovicidal actions have been reported with a high percentage of non-viable *Trichocephalus* eggs after 30 days [10].

In a recent study, Kolodziejczyk et al. (2019) evaluated the potential ovistatic action of soil fungi against *Ascaris suum* eggs [15]. The selected fungi were *Fusarium oxysporum*, *Fusarium sulphureum*, *Fusarium verticillioides*, and *Penicillium expansum*. *Fusarium sulphureum* had the best action [15]. They did not identify any structural changes of the eggs. The ovistatic effect was induced by the metabolites produced by fungal species, namely: patulin, moniliformine, fumonisine, and griseofulvine [15].

Thapa et al. (2015) observed a decrease in the viability of roundworm eggs using *Pochonia chlamydosporia* biotype 10 and *Purpureocillium lilacinum* biotype 251. *Pochonia chlamydosporia* can be used to eliminate potential parasitic elements with infecting capacity, the results of the study indicating a reduction in the viability of *Ascaridia galli*, 64–86% and those of *Toxocara canis*, of 26–67%. The ovicidal effect was lower in the case of *Purpureocillium lilacinum*, the reduction in viability being 15–29% (*Ascaridia galli*) and 4–28% (*Toxocara canis*). *Ascaris suum* eggs were resistant to fungal action [16]. 

In the case of equine roundworms (*Parascaris equorum*), several genera of ovicidal fungi have been identified: *Fusarium*, *Lecanicillium*, *Mucor*, *Trichoderma*, *Verticillium*, *Penicillium*, and *Gliocladium*. The first five listed genera also caused damage to the internal structures of the eggs following the *hyphal* adhesion to the egg wall and the gradual colonization, continued by the complete destruction of the embryo [17].

Similar structural changes have been observed by other researchers in certain studies involving the action of fungi such as *Chrysosporium indicum* and *Chrysosporium keratinophylum*. These two species can alter the membrane, but also the internal content of *Toxocara canis* eggs and, thus, can be used in the control of ascariosis in animals [18].

Under optimal conditions (a constant temperature of 28 °C and a continuous, daily aeration for 30 min), the internal cell division, in the case of *Toxocara canis* eggs, begins within 24 h of exposure, and in 3–5 days, most evolutionary stages can be observed (one cell, two cells, three cells, four cells, morula, blastula, gastrula, etc.), except for the larval stages. The larvae can generally be seen from the 14th day [2,19] onwards. The thermal values recorded throughout the study period can be associated with those periods of the year which ensure the best conditions for the development of parasitic elements. These thermal differences are also observed in natural conditions, in the external environment, in western Romania.

Some studies indicate an optimal range of relative humidity between 23 and 50% [2]. Thus, the values of relative humidity, recorded throughout the present study, provided adequate conditions for the development of parasitic elements.

No biological control research, with the use of certain fungi against *Toxocara cati* eggs, have been found in the literature. Thus, the present study is a starting point for further research in the field.

Only the direct (ovicidal) effect of Ecosin^®^ was investigated, following the application protocol indicated in the package leaflet. However, according to the results, differences were observed in the number of viable eggs that evolved at different stages. The percentages were lower in the case of samples exposed to oomycete. This may suggest a potential ovistatic action induced by *Pythium oligandrum*. The short period of time chosen for conducting the research (according to the manufacturer) does not fully explain this phenomenon. According to a recent study from 2020 [20], in the case of *A. suum*, L1 is formed inside the eggs at 14 days and L2 after about 21 days. The highest percentage of eggs with L2 appears after 28 days. 

It is necessary, in the future, to carry out studies for a longer period to confirm the ovistatic action. Thus, the protocol and the total period of application must be readjusted to be able to identify the best control method.

## 4. Materials and Methods

### 4.1. Egg Collection

The canine and feline patients, that were brought in for consultation at the Parasitology Clinic from the Faculty of Veterinary Medicine, accusing symptoms that were typical for *Toxocara* spp. infection, were tested and the positive cases received proper deworming treatment. 

Following the administration of the nematicidal treatment, some of the owners agreed to bring back the *Toxocara* spp adults identified in the feces of their dogs. The animals were dewormed with Caniverm^®^ (Bioveta, Komenského, Czech Republic), a product containing fenbendazole, prazicuantel and pyrantel embonate. Two administrations were used, at 14 days intervals between them. After each administration, the adults excreted in the feces were brought to the clinic within 24 h for processing. They were placed in plastic containers with saline solution to prevent rapid dehydration.

Ascarid females were selected and sectioned longitudinally to identify the uterine loops. According to Lysek and Ondrus (1992) [21], the O-U junction of the uterus was microscopically identified. At this level the complete fertilization of the eggs takes place along with the formation of all the covering layers of the egg wall. The uterine loops were sectioned posterior to this junction, in order to obtain eggs that have a completely developed wall. These were, in turn, ground and filtered (0.5 mm sieve) to collect the eggs. Approximately 12,000 *Toxocara canis* eggs and 12,000 *Toxocara cati* eggs were obtained (McMaster technique).

All the selected eggs were undeveloped at the beginning of the study.

### 4.2. Sampling, Analysis and Preparation of Soil Samples

The soil samples were collected from various dog enclosures using a probe. The probe was introduced at a 10 cm depth. The method described by Gee and Bauder (1986) [22] was used to identify and classify the soil types. 

Currently, there are no pure soils on the field but technosols. These are a combination of at least two types of soil.

The soils in which the sand is found have a higher drainage ability, are not sticky (very compact) and retain little water. 

Clay soils are more compact and stickier. They are difficult to drain and retain large amounts of water.

Clay soils are rich in organic matter.

The soil samples were weighed using the analytical balance and 50 g of soil were distributed in 90 mm × 15 mm Petri dishes. These were autoclaved (150 °C, 30 min) to obtain sterile media [23].

A total of 100 samples was obtained:

- *n* = 50 (Petri dishes with *Toxocara canis* eggs and soil): *n* = 25—samples exposed to Ecosin^®^ (*n* = 5—samples for each soil type) and *n* = 25—control samples;
- *n* = 50 (Petri dishes with *Toxocara cati* eggs and soil): *n* = 25—samples exposed to Ecosin^®^ (*n* = 5—samples for each soil type) and *n* = 25—control samples.

### 4.3. Preparation of Sandstone Media

Twenty tiles cut at 10 cm × 10 cm were prepared in order to imitate the paddocks from the dog shelter. They were separated using cardboard walls.

A total of 20 samples was obtained:

- *n* = 10 (*Toxocara canis* eggs): *n* = 5—samples exposed to Ecosin^®^ and *n* = 5—control samples;
- *n* = 10 (*Toxocara cati* eggs): *n* = 5—samples exposed to Ecosin^®^ and *n* = 5—control samples.

### 4.4. Preparation of Pythium oligandrum Solution

The product used in this study was Ecosin^®^ (Maravet, Galenicka laboratory Ostrava, Obrancu miru 234/41, 703 00 Ostrava-Vitkovice, Czech Republic), containing the following ingredients: citric acid, baking soda, sorbitol, silicon dioxide, *Panicum miliaceum*, *Pythium oligandrum* (oospores), PEG 6000, and sodium carbonate. A solution was prepared according to the instructions found in the product package. Thus, one tablet (3 g) was dissolved in 2 L of lukewarm water (34 °C).

### 4.5. Egg Exposure

Approximately 200 *Toxocara* spp. eggs were added to each soil sample in an even layer. The eggs were evenly distributed on the tiles. In order to maintain a moist environment and prevent desiccation, they were sprayed with water three times a day for seven days. The soil samples benefited from the same water-spraying treatment during the entire study period.

The 10 mL of the prepared solution was sprayed on each medium. Due to inactivation of the product within 24 h, the solution was applied three times every two days. Saline solution was used instead of Ecosin^®^ in the control samples. After seven days, 100 eggs were examined from each sample. Soil samples were examined using a combined sedimentation and flotation method. Thus, about 30 g were weighed on the analytical balance and homogenized in a beaker with distilled water (28 mL) and two drops of Tween^®^ 20 (Sigma-Aldrich, Saint Louis, MO, USA) solution for about 5 min. The obtained content was drained through a sieve, into a beaker and then placed in two 14 mL tubes which were centrifuged at 3000 rpm for 10 min. The supernatant was removed and supersaturated solution was added instead. The contents of each tube were examined using the Willis method [24]. 

In the case of sandstone media, they were initially sprayed with water and the contents (approximately 5 mL) were collected with a pipette and examined using the Willis method [24].

The ascarid eggs were examined under a Leica DM2500 microscope (10×, 40× obj.) and were divided into two categories (viable and non-viable) to assess the ovicidal action of the product. According to Gautam et al. (2014), the viable eggs were the larvated, the undeveloped, or the ones in various evolutionary stages, while the non-viable eggs were the eggs showing degenerative changes [25].

The temperature (minimum and maximum) and relative humidity of the environment were monitored throughout the seven experimental days, using the indoor weather station, Konig LCD Weather Station KN-WS102N. A daily light/dark cycle was provided during the study: 10/14 h.

### 4.6. Statistical Interpretation

The obtained results were statistically interpreted using a Mann–Whitney test in the GraphPad Prism program. The value of *P* was determined to assess the degree of significance.

## 5. Conclusions

The oospores of *Pythium oligandrum*, contained by the Ecosin^®^ product, have an ovicidal effect on *Toxocara canis* and *Toxocara cati* eggs. Thus, the oomycete can be used to decontaminate different type of soils and various surfaces where these parasitic elements are found.

## Figures and Tables

**Table 1:** Ecosin^®^ action against *T. canis* eggs at 7 days.

Sample	Media	Viable Eggs			Non-Viable Eggs	
		% Larvated (am)	% Developed (Prelarval Stages) (am)	% Undeveloped (am)	% Degenerate (am)	Mann-Whitney Test (*p* Value)
treated (*n* = 5)	sandy-clayey clay	0	43.8	21.2	35	0.0079 (**)
control (*n* = 5)	sandy-clayey clay	0	67.4	29.2	3.4	
treated (*n* = 5)	medium clay	0	32.8	44.6	22.6	0.0159 (*)
control (*n* = 5)	medium clay	0	63.4	26.2	10.4	
treated (*n* = 5)	medium sandy clay	0	22.8	38.2	41	0.0079 (**)
control (*n* = 5)	medium sandy clay	0	65.2	23.8	11	
treated (*n* = 5)	loamy fine sand	0	48.6	31.6	21.8	0.1032 (ns)
control (*n* = 5)	loamy fine sand	0	69	20.6	10.4	
treated (*n* = 5)	coarse sandy clay	0	49.8	32.4	17.8	0.0952 (ns)
control (*n* = 5)	coarse sandy clay	0	69.2	25.2	5.6	
treated (*n* = 5)	sandstone media	0	45.2	33.8	21	0.0079 (**)
control (*n* = 5)	sandstone media	0	71	19.6	9.4	

am = arithmetic mean, ns—not significant, *—significant, **—very significant.

**Table 2:** Ecosin^®^ action against *T. cati* eggs at 7 days.

Sample	Media	Viable Eggs			Non-Viable Eggs	
		% Larvated (am)	% Developed (Prelarval Stages) (am)	% Undeveloped (am)	% Degenerate (am)	Mann-Whitney Test (*p* Value)
treated (*n* = 5)	sandy-clayey clay	0	37.6	27.4	35	0.0079 (**)
control (*n* = 5)	sandy-clayey clay	0	67.6	20	12.4	
treated (*n* = 5)	medium clay	0	30.2	48.6	21.2	
control (*n* = 5)	medium clay	0	69.6	20.2	10.2	
treated (*n* = 5)	medium sandy clay	0	29	24.6	46.4	
control (*n* = 5)	medium sandy clay	0	66.8	21.2	12	
treated (*n* = 5)	loamy fine sand	0	39.2	30.8	30	
control (*n* = 5)	loamy fine sand	0	69.2	21.2	9.6	
treated (*n* = 5)	coarse sandy clay	0	39	30.8	30.2	
control (*n* = 5)	coarse sandy clay	0	67.6	24.4	10	
treated (*n* = 5)	sandstone media	0	37.6	31.4	31	
control (*n* = 5)	sandstone media	0	68.6	22.8	8.6	

am = arithmetic mean, **—very significant.
